# Hydrogen Bond-Mediated Conjugates Involving Lanthanide Diphthalocyanines and Trifluoroacetic Acid (Lnpc_2_@TFA): Structure, Photoactivity, and Stability

**DOI:** 10.3390/molecules25163638

**Published:** 2020-08-10

**Authors:** Gabriela Dyrda, Maja Zakrzyk, Małgorzata A. Broda, Tomasz Pędziński, Giuseppe Mele, Rudolf Słota

**Affiliations:** 1 Faculty of Chemistry, University of Opole, Oleska 48, 45-052 Opole, Poland; maja87z@gmail.com (M.Z.); broda@uni.opole.pl (M.A.B.); rslota@uni.opole.pl (R.S.); 2 Faculty of Chemistry, Adam Mickiewicz University in Poznań, ul. Uniwersytetu Poznańskiego 8, 61-614 Poznań, Poland; tomek@amu.edu.pl; 3 Department of Engineering for Innovation, University of Salento, Via Arnesano, 73100 Lecce, Italy; giuseppe.mele@unisalento.it

**Keywords:** lanthanide diphthalocyanines, TFA conjugates, photostability, UV-Vis spectra, singlet oxygen, photon upconversion

## Abstract

The interaction between lanthanide diphthalocyanine complexes, LnPc_2_ (Ln = Nd, Sm, Eu, Gd, Yb, Lu; Pc = C_32_H_16_N_8_, phthalocyanine ligand) and trifluoroacetic acid (TFA) was investigated in benzene, and the stability of the resulting molecular system was assessed based on spectral (UV-Vis) and kinetic measurements. Structural Density Functional Theory (DFT) calculations provided interesting data regarding the nature of the bonding and allowed estimating the interaction energy between the LnPc_2_ and TFA species. Conjugates are created between the LnPc_2_ and TFA molecules via hydrogen bonds of moderate strength (>N∙∙H··) at the *meso*- -bridges of the Pc moieties, which renders the sandwich system to flatten. Attachment of TFA is followed by rearrangement of electronic density within the chromophore system of the macrocycles manifested in considerable changes in their UV-Vis spectra and consequently the color of the studied solutions (from green to orange). The LnPc_2_@TFA conjugates including Nd, Sm, Eu, and Gd appeared evidently less photostable when exposed to UV radiation than the related mother compounds, whereas in the case of Yb and Lu derivatives some TFA-prompted stabilizing effect was noticed. The conjugates displayed the capacity for singlet oxygen generation in contrast to the LnPc_2_s itself. Photon upconversion through sensitized triplet–triplet annihilation was demonstrated by the TFA conjugates of Nd, Sm, Eu, and Gd.

## 1. Introduction

**Figure 1:**
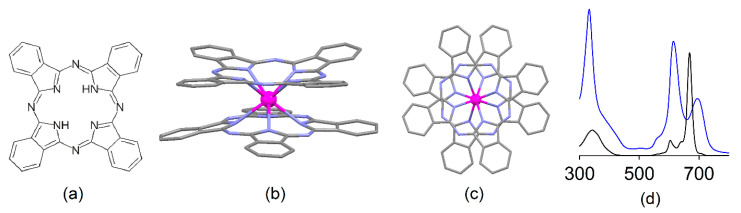
Free-base phthalocyanine, H_2_Pc (**a**); general molecular structure of LnPc_2_ (**b**) and top view (**c**); electronic absorption spectra of LuPc_2_ (blue line) and ZnPc (black line) in DMF (**d**).

Phthalocyanine derivatives are ranked among one of the most important molecular materials. From among a great number of possible structural variations, particularly the sandwich complexes of the lanthanide metals series offer a wide range of useful features meeting the demands for smart chemical components to be used for versatile purposes [1,2]. These include essentially modern electronics, optoelectronics, and catalytic applications. The peculiar molecular system consisting of a trivalent lanthanide (Ln) ion coordinated by two Pc macrocycles (Figure 1) renders the LnPc_2_ semiconductors display a considerably smaller energy bandgap and hence much better electrical conductivity and higher charge mobility compared with typical metallophthalocyanines (e.g., CuPc, ZnPc) [3,4]. This is considered crucial for intermolecular charge transfer in film integrated circuits, catalytic systems, and photoactive composites. Moreover, the LnPc_2_s effectively absorb the photonic energy from the UV and red-edge range which may boost the electron transfer in photo-conducting electronic components.

Generally, the phthalocyanines are considered relative stable under visible and infrared light but they exhibit diverse resistance to UV radiation [5,6]. Among them, the LnPc_2_ complexes featured remarkable photostability in organic media [7,8].

The phthalocyanine macrocycle has been proved susceptible to interactions with several diverse molecules via the *meso*-nitrogen atoms (N-bridges), and particularly with electron acceptor species [9,10,11]. This is a common feature of both the free-base H_2_Pc and its metal complexes, which is considered critically important for application purposes. The most extensive investigations so far referred to the effect of acidic compounds on the chemistry of phthalocyanines while in solution (organic solvents, water), and especially the protonation process was widely explored. It was found that under typical conditions addition of acid usually resulted in protonation only of the four *meso*-N atoms [12,13]; however in the case of H_2_Pc it was suggested elsewhere that possibly the both remaining pyrrole nitrogens may also be engaged [14]. Obviously, the latter one does not apply to the metal complexes.

The protonation process itself is generally regarded as a successive accommodation of the H^+^ species which consequently affects the distribution of electronic density within the inner chromophore system (phthalocyanine core) followed by considerable changes of photochemical properties [15,16]. This is clearly demonstrated in the UV-Vis spectrum by stepwise modification of the Q-band character while increasing the acid concentration in the phthalocyanine solution. Hence, spectrophotometric titration has been widely used as a convenient procedure providing evidence for the formation of mono-, di-, tri- and tetra-cations [15,17,18,19,20,21,22]. The protonation process was found solvent-specific and depends on the stability of the phthalocyanine complex as well as on the strength of the acid used. Basically, the protonated Pc macrocycle is considered less chemically stable [23,24,25,26]. Notably labile compounds (e.g., PbPc, MgPc) do not create stable protonated forms and are subject to prompt demetallation and subsequent cleavage of the Pc moiety, particularly in the presence of strong acids such as H_2_SO_4_ [27,28]. Incidentally, some complexes (NiPc, CuPc) proved to resist even that strong acids, nevertheless several phthalocyanines appeared quite stable in less aggressive protic media, and demonstrated interesting acid-base chemistry [29,30,31,32,33,34,35,36,37,38,39,40,41]. This is regarded as an important quality which determines the wide spectrum of possible application of phthalocyanines as active sensors of protic acids and equally of other electron acceptors, as well as components of semiconductor systems, catalysts and photosensitizers [42]. The lanthanide diphthalocyanines reveal a particular attractive behavior when treated with common Lewis acids, featured by distinct stepwise color changes, both in the solid state and in solution, strictly related to the acid concentration. The effect of both protic and non-protic electron acceptors was reported elsewhere, e.g., acetic acid [43], triflic acid [44], sulfur dioxide [45,46,47] or thionyl chloride [48].

TFA is an important versatile acid (less oxidizing than H_2_SO_4_) used as a reagent in organic synthesis, as an ion pairing agent in HPLC or solvent for NMR spectroscopy, and calibrant in mass spectrometry. It is also extensively used in studies concerning the acidic activation of porphyrins or typical mono-phthalocyanines [26,49,50]. However, in this scope the lanthanide diphthalocyanines thus far were not sufficiently explored. Such activation is often required i.a. in hybrid photocatalytic systems involving phthalocyanine photosensitizers, and usually sulfuric acid has been used to this end [51,52]. Nevertheless, our recent investigations proved that TFA might have appeared yet more effective in enhancing the photochemical properties of LnPc_2_ complexes. For this reason, in the present work we have studied the impact of TFA species on selected diphthalocyanines including the trivalent ions of Nd, Sm, Eu, Gd, Yb, and Lu. To avoid unwanted solvent effects our experiments were carried out in non-polar and neutral benzene. DFT calculations helped assessing structural consequences resulting from the interaction of the *bis*-macrocyclic molecular system with TFA. Photostability of such conjugates (adducts) was the crucial element of this research, since they are expected to show catalytic and biochemical activity, which would make them particularly attractive for light-sensitized medical therapies [53,54,55]. Also, the singlet oxygen issue was relevant here, since its production may somewhat affect the robustness of the phthalocyanine macrocycles toward oxidative degradation [5,6,56,57,58].

## 2. Results and Discussion

### 2.1. Formation of LnPc_2_@TFA Conjugates

**Figure 2:**
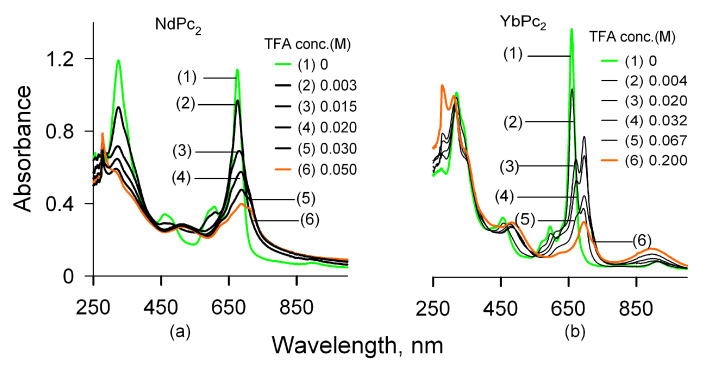
Formation of the TFA conjugates (orange form) with (**a**) NdPc_2_ and (**b**) YbPc_2_ traced by spectrophotometric measurements in the UV-Vis range. The TFA concentration necessary to produce the maximum amount of the Yb-conjugate is 4 × higher than in the case of Nd.

**Figure 3:**
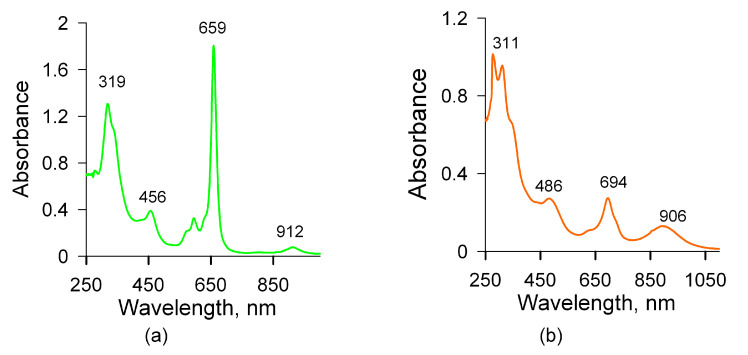
Terminal UV-Vis spectra in benzene of (**a**) YbPc_2_ (green form), and (**b**) YbPc_2_@TFA (orange form).

The interaction between LnPc_2_ and TFA molecules proceeds similar to a typical titration process and finally results in formation of phthalocyanine-acid conjugates, LuPc_2_@TFA. This general formula refers to a double-decker system hosting eight TFA molecules showing up a unique hydrogen bond-mediated set-up (see Section 2.2). The reaction progress can be followed by monitoring the spectral changes in the UV-Vis range, as reported in Figure 2, which are featured by gradual shift in color from the initial green to the eventual orange. These different colored forms represent the two terminal varieties of the same LnPc_2_ unit, characterized by diverse distribution of the delocalized electronic charge within the macrocycle’s chromophore system (phthalocyanine core), Figure 3. The spectral correlation between the mother compound and the orange conjugate reflects the susceptibility of the internal π-electronic core to be effectively affected by polarizing action of electron acceptor species [45] or the electric field potential [1,2]. In the studied case, the reason of the observed changes is the immediate interaction of the proton donor (TFA) with the *meso*-N-bridges of the macrocycle, which may be considered in terms of a *soft oxidizing effect* [7,45].

**Figure 4:**
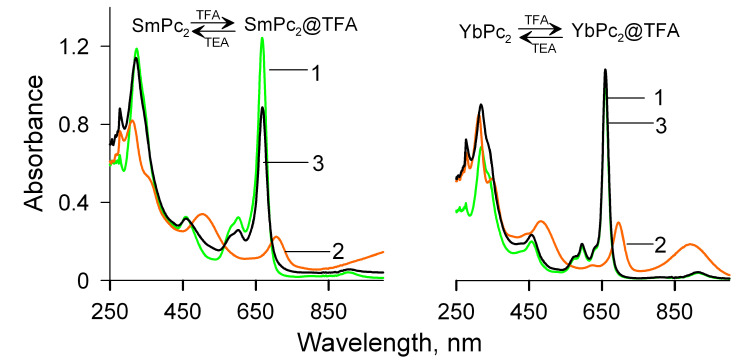
Reversibility of the TFA-conjugate formation: (1) initial form LnPc_2_ (green line), (2) the conjugate LnPc_2_@TFA (orange line), (3) recovery of the initial form after adding TEA to the (2) solution (black line); note the partial decay of the SmPc_2_ molecular system (**left**) and complete regeneration of the YbPc_2_ initial form (**right**).

The TFA quantity required to achieve the maximum amount of the orange conjugate in the benzene solution was found equal for the complexes of Sm, Eu, Gd, Yb, and Lu, and for the initial LnPc_2_ concentration of ca. 2.0∙10^−5^ M the experimentally determined concentration of TFA was 0.2 M. Further addition of the acid (up to 0.3 M) did not increase the amount of LnPc_2_@TFA (i.e., the absorbance of the orange form remained constant). A notable exception proved NdPc_2_ which already at TFA concentration of about 0.05 M produced the orange form, but at the same time showed also apparent symptoms of molecular decay, Figure 2; Figure 4, and Supporting Information (SI) Figures S3 and S4. This follows from the weaker interaction of the Nd^3+^ ion with the both phthalocyanine macrocycles, compared with the heavier more strongly polarizing Ln^3+^ ions, which makes the NdPc_2_ molecules much more susceptible to protonation by TFA than the remaining investigated complexes.

The analysis of the UV-Vis spectra provided some interesting reflections. Since the positions of the B and Q bands were specifically related to the particular phthalocyanines, also the conjugates’ spectra differed from each other, as reported in Table 1 (for more extensive spectral data see SI Figures S1 and S2). Obviously, the observed changes clearly indicate for effective structural modifications involving the macrocycles (see Section 2.2).

In the case of the LnPc_2_@TFA systems, the corresponding B band moved slightly towards shorter wavelengths, whereas the Q-band shifted much more pronouncedly into the red spectral range. Such changes result from the decrease in electron density at the *meso*-N-bridges and consequently in the both Pc chromophores due to the polarizing impact of the TFA protons.

Also, the less intense CT (*charge transfer*) bands may be related to the redistribution of electronic density within the macrocycles. The MLCT (metal-to-ligand CT) bands, upon protonation shifted at the longer wavelengths, and the most pronounced change, 53 nm, was found in the case of NdPc_2_@TFA and the least one, only 25 nm, showed the LuPc_2_@TFA conjugate. This is consistent with the lower polarizing potential of the Nd^3+^ ion compared with that of the Lu^3+^. However, the most significant effect was demonstrated by the LMCT (ligand-to-metal CT) bands, which for the LnPc_2_ complexes fall into the NIR spectral region. Protonation of the macrocycles causes a considerable band shift further into the NIR range only for the conjugates of the Nd (142 nm), Sm (154 nm), Eu (169 nm) and Gd (85 nm) complexes, whereas for the Yb and Lu derivatives the changes were reversed, i.e., the LMCT band appeared at wavelengths shorter of 6 and 23 nm, respectively. This again may be attributed to the stronger interaction of the heaviest lanthanides (Yb, Lu) with the macrocycles hosting four TFA molecules each, demonstrated also by enhanced stability of the YbPc_2_@TFA and LuPc_2_@TFA molecular systems, as followed from photostability studies reported in Section 2.4.

The process of formation of the TFA conjugates was found reversible and the initial LnPc_2_ form may be recovered after introducing of some electron donors into the system, e.g., by passing NH_3_ through the solution or by an addition of triethylamine (TEA). This would have indicated for a moderate interaction strength between TFA and the macrocycles. The reaction of the conjugates with TEA (Figure 4) revealed the strengths and the weaknesses of the individual LnPc_2_ systems, highlighting the significant role of the complexed metal, Table 2.

As already seen during the protonation process, NdPc_2_ represents the least stable compound here. It is generally known that the heavier rare earth elements produce more stable sandwich phthalocyanines than those from the beginning of the row. This was clearly demonstrated by the Yb and Lu complexes which did not suffer from the interaction with TFA and therefore could be regained without any loss after the reaction of their conjugates with triethylamine.

### 2.2. Molecular Structure of the LnPc_2_@TFA Conjugates (DFT Approach)

**Figure 5:**
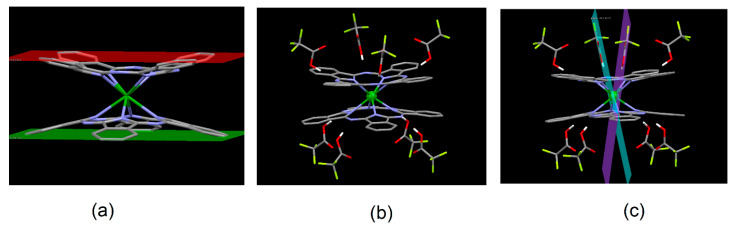
Calculated molecular structures: (**a**) LuPc_2_ featuring the virtual 8C_b_ planes; (**b**) the LuPc_2_@TFA conjugate; (**c**) LuPc_2_@TFA featuring the torsion angle (γ) between the opposite TFA molecules.

The results of DFT computations reported below have been based on the LuPc_2_ and LuPc_2_@TFA model systems, which we believe sufficiently well reflect the main structural implications resulting from simultaneous interaction of the mother LnPc_2_ compound with eight TFA species. This approach allowed also estimating the intermolecular interaction energy (Δ*E*) between the phthalocyanine moiety and TFA. The particular structures have been shown in Figure 5, and the principal molecular parameters have been displayed in Table 3.

The TFA molecule acts as a proton donor linked with the Pc moiety via the *meso*-N atoms (>N_µ_⸱⸱H−OOCF_3_) thus creating a unique hydrogen bond-mediated molecular system LuPc_2_(TFA)_8_, as shown in Figure 5b. The computed interaction energy totaled 245 kJ/mol, hence it was 30.6 kJ/mol per one H-bond, and the mean N_µ_⸱⸱H distance was 180 pm, which indicates for a hydrogen bond of moderate strength (Table 3). Therefore, the acid species can be relatively easy detracted from the conjugates by triethylamine, as demonstrated above (Figure 4). The TFA molecules did not particularly affect neither the Lu−N_p_ distance nor the Pc-core size (S_c_); however, it drastically influenced the periphery of the macrocycles. As follows from Table 3, the interplanar spacing between the cores of the macrocycles (4N_p_, 4N_µ_) actually did not change, which means the structure of the chromophore system remained intact. In fact, the both macrocycles in LuPc_2_@TFA evidently got flattened following the decrease in tilt (14.0° → 8.0°) of the peripheral benzene rings (8C_b_). This resulted in closer distance between the outer virtual planes involving the 8C_b_ atoms in the particular macrocycles (Figure 5a) and certainly produced additional stress in the bonding system. The TFA species, as viewed from their symmetry plane including the C−C axis and the O atoms, are not perpendicular to the macrocycle plane (as found e.g., in ZnPc@TFA) and hence the torsion exhibited by the opposite TFA molecules (Figure 5c). Indeed, these structural effects well correspond with the observed spectrochemical properties reported in Section 2.1. Further DFT studies concerning other LnPc_2_ conjugates are in progress now and will be reported in a successive work.

### 2.3. Fluorescence Spectra and Singlet Oxygen Generation

**Figure 6:**
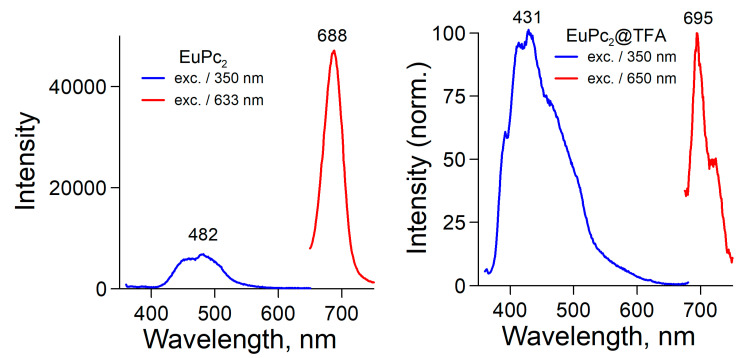
Fluorescence emission spectra of the base EuPc_2_ complex (**left**) and its TFA-conjugate (**right**) in benzene solution.

Both LnPc_2_ and LnPc_2_@TFA produced fluorescence emission once excited by photons of energy matching up the B or Q absorption bands, Figure 6 (SI, Figure S6). Most of the investigated compounds displayed intensive blue emission. On the other hand, the fluorescence in the red range proved to be pronounced only in the case of the LnPc_2_ solutions, whereas the LnPc_2_@TFA conjugates showed a very weak emission, except for NdPc_2_@TFA.

Fluorescence lifetimes were calculated from the quenching curves measured after excitation using a 633 nm laser beam. No peculiar relationship to the investigated LnPc_2_ compounds was found and the obtained values were on the nanosecond timescale, in the range of 4.5 ns (NdPc_2_) to 5.8 ns (YbPc_2_). However, in case of the TFA conjugates the fluorescence decay kinetics appeared more complicated, and the lifetimes could be estimated less precisely than for the base complexes. Nevertheless, these values appeared definitely smaller, approximately between 2.3 ns (YbPc_2_@TFA) and 2.8 ns (SmPc_2_@TFA).

**Figure 7:**
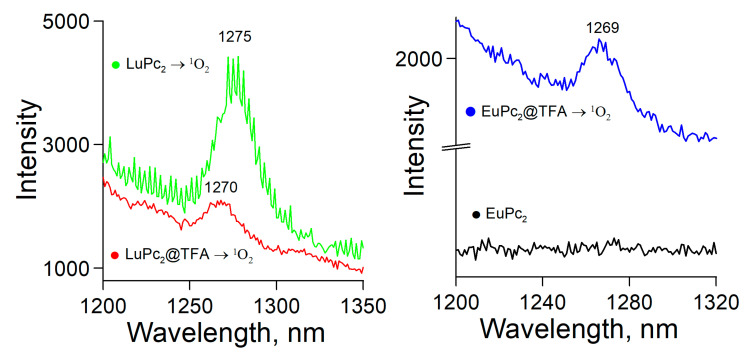
Singlet oxygen (^1^Δ_g_) NIR emission spectra measured in benzene for LuPc_2_, EuPc_2_ and their TFA conjugates (λ_exc_ = 690 nm) due to ^1^Δ_g_ → ^3^Σ^−^_g_ transition (Scheme 1).

The Stokes shift (Table 4) was large for excitations in the shortwave range, Δ_S_(B), which means that radiationless emission contributed considerably to the quenching of the excited states. On the other hand, excitation in the longwave range resulted in much smaller Stokes shifts featuring Δ_S_(Q) of 19–35 nm, but only in the case of LnPc_2_ base compounds. It must be noted that they were evidently greater for the heavier metals. Interestingly, the LnPc_2_@TFA conjugates yielded anti-Stokes shifts (Δ_S_ < 0), which is indicative of photon upconversion. In such case the excitation by lower-energy photons gives rise to photons of higher energy. This phenomenon may be explained in terms of a sensitized triplet–triplet annihilation nonlinear process [59]. The singlet excited state of metal phthalocyanines may be converted into the triplet state (intersystem crossing) and this process is especially effective for closed-shell heavy metal ions which strongly enhance the spin-orbit coupling and hence the population of triplet excited states. Indeed, all the studied TFA conjugates revealed the potential to produce triplet excited states, which was confirmed by their capability to generate singlet molecular oxygen (^1^Δ_g_), Figure 7. Hence, such molecules may also have actively promoted the photon upconversion process. This particular feature is very important i.a. in converting longwave red or NIR light into more energetic photons for the reason of smart optoelectronic devices and particularly operational photovoltaics [59]. The LnPc_2_-based conjugates involving diverse electron acceptor species represent a group of prospective photoactive molecular systems and they will be dedicated a more detailed photochemical investigation in an upcoming project.

**Scheme 1:**
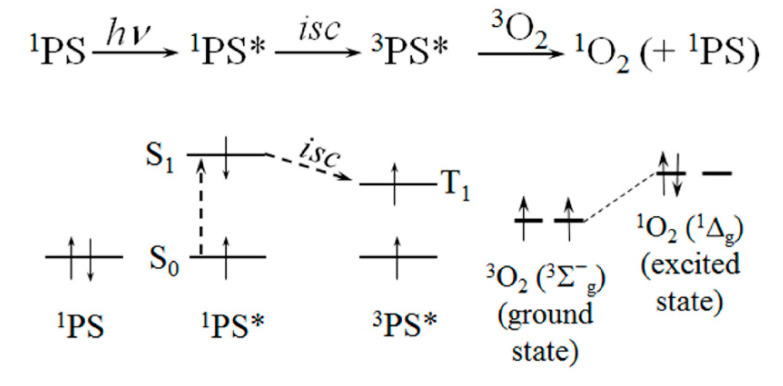
Basic photoreaction path resulting in generating of singlet molecular oxygen (^1^Δ_g_) involving a phthalocyanine photosensitizer PS (top) and simplified energetic diagram (below) showing the excitation of the PS from the singlet ground state S_0_ into the excited singlet S_1_ state next converted into the triplet excited state T_1_ (*intersystem crossing, isc*); the energy of the T_1_ state (of the ^3^PS*) is then transferred to the oxygen molecules (while in their triplet ground state, ^3^O_2_) thus promoting the creation of singlet oxygen species (^1^O_2_).

Of all the investigated LnPc_2_ systems, only the complex of the closed-shell Lu^3+^ effectively generated singlet oxygen (^1^Δ_g_), whereas this was typical for each of the tested TFA conjugates. This was confirmed by a characteristic radiative emission at 1270 nm, following the collisional triplet–triplet energy transfer from the excited photosensitizer (phthalocyanine) to the ground triplet state of molecular oxygen, Figure 7 (Figure S7). The singlet oxygen lifetimes determined for the respective conjugates were on the microsecond timescale, within the range of 21.6 µs (SmPc_2_@TFA) and 27.3 µs (YbPc_2_@TFA). It is commonly known that under certain conditions the photoexcited phthalocyanines may also populate the triplet state which may further be quenched by triplet-state acceptors such as O_2_ molecules, thus yielding the ^1^Δ_g_ singlet oxygen. A simplified reaction path and a related energetic diagram have been shown in Scheme 1. It is not quite clear, what is the principal root of this peculiar property in the case of the LnPc_2_@TFA conjugates, though undoubtedly protonation of the *meso*-N atoms of the Pc macrocycles apparently favors the intersystem S_1_ → T_1_ transition. Some mechanistic aspects referred to this point must still be elucidated, and further research needs to be carried out to encompass this problem.

### 2.4. Photostability

**Figure 8:**
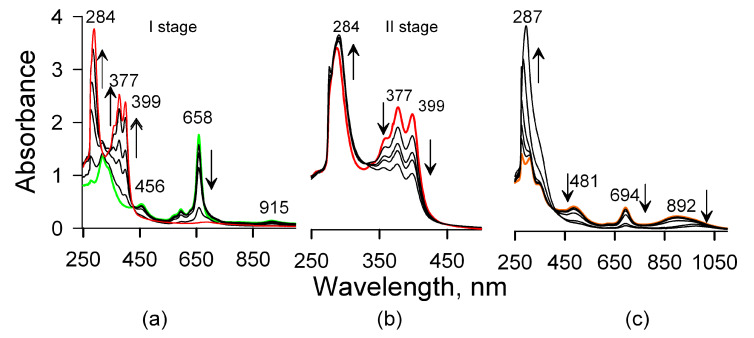
Photodegradation of LuPc_2_ and its TFA-conjugate in benzene solution upon exposure to UV irradiation; arrows indicate the time-correlated trend in changes of the crucial bands (wavelength specified). (**a**) The I stage of LuPc_2_ photolysis, t = 0 (green line) and t = 400 min (red line); (**b**) The II stage starting from t = 400 min up to 850 min; (**c**) Course of the LuPc_2_@TFA photolysis, t = 0 (orange line) up to 300 min.

All the investigated systems proved susceptible to UV irradiation. Photodegradation of LnPc_2_ molecules has developed according to a two-step mechanism, Figure 8. The process was found irreversible and the spectral changes observed during the I stage correspond to the decay of the sandwich system and gradual cleavage of the Pc macrocycles. Meanwhile, an intermediate product has been formed, reaching its maximum concentration after the complete decomposition of the starting compound, Figure 8a. In the II stage further degradation of this product has been continued. On the other hand, photodegradation of the LnPc_2_@TFA conjugates went smoothly on in one step only, Figure 8c. More photodegradation related spectra have been shown in SI, Figure S8. Incidentally, the main degradation product seems to be equal in all studied cases, and based on the UV-Vis and FTIR analyses of the post-reaction residue most likely it is phthalimide (SI, Figure S10a,b), often found in photolyzed phthalocyanine solutions [7]. This would indicate for an oxygen-mediated photodegradation process.

The kinetic profiles based on absorbance (A) measurements, A = f(t), determined for the photodegradation of LnPc_2_ (stage I and II) and LnPc_2_@TFA, all followed the simple first-order exponential relation, A = A_0_ × e^−kt^ (where A_0_ = A_(t = 0)_). The effective rate constants, k, have been shown in Table 5 and the related kinetic curves are reported in SI, Figure S9. However, the quantum yields (Φ) of the photodegradation process could only be roughly estimated based on the kinetic data, and as such they have not been considered relevant for the discussion here. Nevertheless, they have been reported with some comments in the Supplementary Information section (Table S1). Incidentally, these results perfectly align with the kinetic data presented below.

As follows from the kinetic data, in essence, the decay of the sandwich system in LnPc_2_ has been accomplished approximately within the first 400 min of UV-illumination (SI, Figure S8). The most UV-resistant proved the lutetium compound, whereas the other ones surprisingly including also YbPc_2_ showed lower (but alike) stability. The II stage evidently applies to the photodecomposition of the same kind of intermediate product; hence the reaction rates are roughly of similar order. Protonation by TFA considerably weakened the molecular stability in the case of the Nd, Sm, Eu, and Gd compounds, and particularly that of the NdPc_2_@TFA conjugate, which has supported the results discussed in Section 2.1. Interestingly, the chromophore systems in the Yb and Lu conjugates appeared definitely more photostable than they showed before protonation. Since the observed photodegradation is actually realized due to the reaction of the photoexcited phthalocyanines with oxygen species first at the N-bridges integrating the macrocycle system, the stabilizing back-polarizing effect of the metal ion and some shielding provided by the hosted TFA molecules may be crucial to this point. This might have explained the increased photostability of the systems including the stronger polarizing Yb^3+^ and Lu^3+^ ions. In contrast to the latter ones, the other lanthanide ions evinced less potential to effectively cope with the impact of TFA (reducing the electronic density on the bridging N atoms) resulting in flattened and hence more stressed macrocycles. However, the role of singlet oxygen ^1^O_2_ in the photodecay of these compounds is not quite clear-cut, most probably this reactive species might not had been generated under the conditions of the photostability investigations.

## 3. Materials and Methods

### 3.1. Materials

Diphthalocyanine complexes of Ln(III) (Nd, Sm, Eu, Gd, Yb, and Lu) were synthesized in our laboratory (Institute of Chemistry, University of Opole) according to the own developed procedure [8,60]. Benzene and trifluoroacetic acid (TFA), were purchased from Sigma-Aldrich (Poznań, Poland) and used as supplied.

### 3.2. Spectrophotometric Measurements

JASCO V-670 UV-Vis-NIR Spectrophotometer (Spectra Manager V.2 software) and quartz cuvettes (1 cm optical path length, V = 4 mL) were used to measure electronic absorption spectra. FluoTime 300 fluorescence spectrometer (PicoQuant, Berlin, Germany) was used to collect emission spectral data.

Singlet oxygen (^1^Δ_g_) emission spectra were measured directly (in benzene) according to the procedure reported in [50]. For this reason, a technique of time-resolved near infrared detection of singlet oxygen (O_2_(^1^Δ_g_)) phosphorescence at 1270 nm has been developed using a FluoTime 300 fluorescence spectrometer (PicoQuant, Berlin, Germany) equipped with a picosecond diode laser (440 nm) operating at 40 MHz repetition rate [61]. This procedure allowed distinguishing between a usually “slow” singlet oxygen emission occurring on a microsecond timescale, and a fast, nanosecond room temperature phosphorescence of the sensitizer. The singlet oxygen lifetime (τ_Δ_) was calculated from the phosphorescence decay at λ = 1270 nm (at 22°C).

### 3.3. Phthalocyanine Protonation by TFA

Titration of the respective phthalocyanines in benzene was carried out in a 4 mL quartz cuvette as follows; 1 mL of LnPc_2_ solution (4.0 × 10^−5^ M) was mixed with 1 mL of a benzene solution containing a specified amount of TFA, and hence the initial concentration of LnPc_2_ was kept constant (2.0 × 10^−5^ M) in all samples tested. The resulting solution was agitated, and its UV-Vis spectrum was measured thereon. The TFA concentration was gradually increased, and the procedure was continued until the maximum absorbance of the protonated form, i.e., the orange-colored LnPc_2_@TFA conjugate, was achieved.

### 3.4. Photostability Studies

To assess the photostability a similar procedure was applied, as in our previously reported works [7,8]. The tested solutions were exposed to UV radiation (500 μW/cm^2^) in a thermostated quartz cuvette (at 20°C). For this purpose, a mercury medium pressure lamp (Heraeus St-46) characterized by narrow emission lines of similar spectral irradiance at λ = 313, 366, and 436 nm was used. Irradiance was measured at the cuvette wall facing the light source. The explored solutions were not de-aerated prior to testing. Kinetic curves, *A = f(t)*, reflecting the phthalocyanine degradation were determined from time-correlated absorbance measurements, i.e., from the peak absorption value of the Q-band. The effective photolysis rate constants, *k*, were computed directly from kinetic data (CurveExpert v.1.34 software, Hyams Development, Huntsville, AL, USA).

### 3.5. Theoretical Structural Studies

The DFT approach appeared sufficiently accurate to characterize the modification of the porphyrin’s chromophore system due to the impact of trihaloacetic acids [49,50]. Moreover, it was successfully used to describe the structural and spectroscopic properties of lanthanide complexes [62,63,64], hence in this study we have applied the B3LYP hybrid functional with the 6-31G(d), LANL2DZ, def2-TZVP basis sets for geometry optimizations using standard parameters in the Gaussian 09 program package [65]. Harmonic frequency calculations were performed to assure that the computed structures refer to the true energy minima. Conjugates between the LnPc_2_ and TFA molecules were created via hydrogen bonds at the *meso*-N-bridges of the Pc moieties. All interaction energies were corrected for basis set superposition error (BSSE) applying the counterpoise method [66] as implemented in Gaussian 09.

## 4. Summary

Sandwich complexes of phthalocyanine comprising Ln^3+^ ions readily undergo protonation by TFA, and the process may be reversed by adding of an electron donor. The resulting conjugates represent H-bond-mediated systems in which the macrocycles have been forced to adapt a flatter and hence more stressed structure than in a typical LnPc_2_ unit. Hence, their stability depends on the back-polarizing potential of the coordinated metal, which has manifested in the much more stable structures of Yb and Lu compounds in contrast to the other metals. The LnPc_2_@TFA conjugates demonstrated interesting photochemistry, featuring i.a. photon upconversion capability and potential to generate singlet oxygen, both of which are supposed to attract attention of the scientific community.

## Figures and Tables

**Table 1:** Peak position (λ_max_, nm) of the B, CT (*charge transfer*) and Q bands in the UV-Vis spectra of the initial (green) LnPc_2_ complexes and the LnPc_2_@TFA conjugates (orange) in benzene; MLCT = metal-to-ligand charge transfer, LMCT = ligand-to-metal charge transfer.

Compound Band		NdPc_2_	SmPc_2_	EuPc_2_	GdPc_2_	YbPc_2_	LuPc_2_
LnPc_2_ (green form)	B	324	322	323	322	319	318
	MLCT	461	459	458	458	456	456
	Q	675	669	668	667	659	658
	LMCT	900	901	901	904	912	915
LnPc_2_@TFA (orange form)	B	312	311	311	308	311	311
	MLCT	514	503	509	498	486	481
	Q	710	707	709	708	694	694
	LMCT	1042	1055	1070	989	906	892

**Table 2:** Degradation degree, δ(%), of LnPc_2_ during the reaction of LnPc_2_@TFA with triethylamine (TEA) in benzene; δ = [(A_0_ − A_1_)/A_0_] × 100%, where A_0_ and A_1_ refer to the initial and final absorbance (recovered) of LnPc_2_, respectively (see Figure 4 and Figure S4 in SI).

Compound	NdPc_2_	SmPc_2_	EuPc_2_	GdPc_2_	YbPc_2_	LuPc_2_
δ%	100	28	27	6	0	0

**Table 3:** Selected molecular data for the base LuPc_2_ complex and its LuPc_2_@TFA conjugate; *ΔE*—total intermolecular interaction energy, *S_c_*—macrocycle core perimeter (Ǻ), Lu−N_p_ is the mean metal−N(pyrrole) distance (Ǻ) (standard deviation < 10^−3^); 4N_p_, 4N_µ_ and 8C_b_ refer to the distance between the both macrocycles expressed by interplanar spacing of planes defined by the 4 pyrrole N, 4 *meso*-N and the 8 peripheral benzene C atoms, respectively (Ǻ); N_µ_⸱⸱H is the mean hydrogen bond length (Ǻ) (standard deviation < 10^−3^); α—inclination of the benzopyrrole units toward the 4N_p_ plane; β—inclination of the C−C axis in TFA toward the 4N_µ_ plane; γ—torsion angle between the opposite TFA molecules (see SI Figure S5).

Compound	*ΔE* kJ/mol	Lu−N_p_	S_c_	N_µ_⸱⸱H	Interplanar Spacing			α (°)	β (°)	γ (°)
					4N_p_	4N_µ_	8C_b_			
LuPc_2_	−	2.416	21.602	−	2.735	3.328	5.004	14.0	−	−
LuPc_2_@TFA	245	2.422	21.645	1.800	2.747	3.310	4.123	8.0	32.0	21.4

**Table 4:** Fluorescence emission spectral data; wavelengths (nm) referring to the emission maxima are shown for the base LnPc_2_ compounds and for their TFA conjugates; excitation was performed using laser radiation of 350, 633, and 650 nm; Stokes shift, Δ_S_ (nm), calculated relative to the λ_max_ value of the B and Q bands, Δ_S_(B) and Δ_S_(Q), respectively.

λ_exc_, nm	NdPc_2_	SmPc_2_	EuPc_2_	GdPc_2_	YbPc_2_	LuPc_2_
350	434	435	482	487	430	429
633	694	691	688	695	692	693
Δ_S_ (B)	110	113	159	165	111	111
Δ_S_ (Q)	19	22	20	28	33	35
**LnPc_2_@TFA**						
350	428	471	431	430	430	430
650	695	694	695	693	693	694
Δ_S_ (B)	116	160	120	122	119	119
Δ_S_ (Q)	−15	−13	−14	−15	−1	0

**Table 5:** Effective photolysis rate constants, k (min^−1^), determined for the I stage and LnPc_2_@TFA at the Q-band, and for the II stage at λ = 399 nm.

Photolysis	NdPc_2_	SmPc_2_	EuPc_2_	GdPc_2_	YbPc_2_	LuPc_2_
I stage	1.8 × 10^−2^	1.4 × 10^−2^	2.1 × 10^−2^	1.5 × 10^−2^	1.3 × 10^−2^	4.6 × 10^−3^
II stage	1.7 × 10^−3^	8.0 × 10^−4^	1.2 × 10^−3^	1.4 × 10^−3^	1.8 × 10^−3^	1.3 × 10^−3^
LnPc_2_@TFA	4.4 × 10^−1^	2.8 × 10^−2^	3.0 × 10^−2^	3.0 × 10^−2^	5.6 × 10^−3^	3.7 × 10^−3^

## Supplementary Materials

The following are available online. Extensive absorption and emission spectra, molecular structure details, and kinetic curves are available online.
