# Supplementary material for: Hydrogen Bond-Mediated Conjugates Involving Lanthanide Diphthalocyanines and Trifluoroacetic Acid (Lnpc2@TFA): Structure, Photoactivity, and Stability
Source: Molecules. 2020 Aug 10;25(16):3638. doi: 10.3390/molecules25163638 (PMC7463839; doi:10.3390/molecules25163638)
Supplement: Supplementary file 1 [file molecules-25-03638-s001.pdf]

Hydrogen bond-mediated conjugates involving lanthanide diphthalocyanines and trifluoroacetic acid ($LnPc_2$@TFA): structure, photoactivity and stability

G Dyrda, M. Zakrzyk, M.A. Broda, T. Pędziński, G. Mele, Rudolf Słota

Table of Contents for Supplementary Information data

Page

1. UV-Vis spectra of $LnPc_2$ in benzene ………………………………………………… 2

2. UV-Vis spectra of $LnPc_2$@TFA conjugates in benzene …………………………….. 2

3. The protonation process of $LnPc_2$ complexes ……………………………………….. 3

4. Chemical stability of the $LnPc_2$@TFA conjugates ……………………………………. 4

5. Structural considerations based on the DFT-created model …………………………… 5

6. Fluorescence spectra of $LnPc_2$ and $LnPc_2$@TFA in benzene …………………………. 6

7. Singlet molecular oxygen related NIR emission spectra ………………………………. 8

8. The photodegradation process (UV-Vis spectra) ………………………………………. 9

9. The photodegradation process (kinetic curves) ..……………………………………… 11

10. Quantum yield ……………………………………………………………………... 12

11. The photodegradation product ……………………………………………………...… 12

1. UV-Vis spectra of $LnPc_2$ in benzene

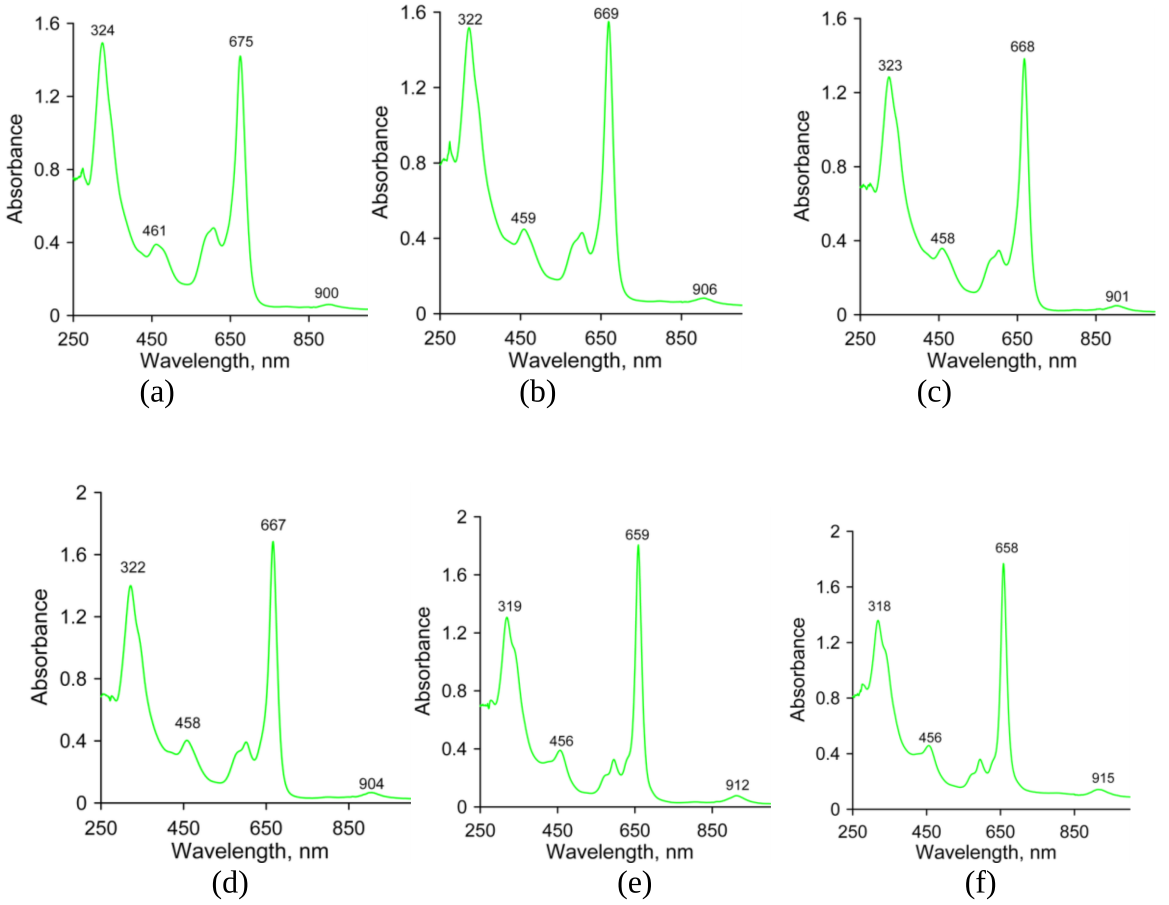

Figure S-1. UV-Vis spectra in benzene of (a) $NdPc_2$, (b) $SmPc_2$, (c)$EuPc_2$, (d) $GdPc_2$, (e) $YbPc_2$, (f) $LuPc_2$.

2. UV-Vis spectra of $LnPc_2$@TFA conjugates in benzene

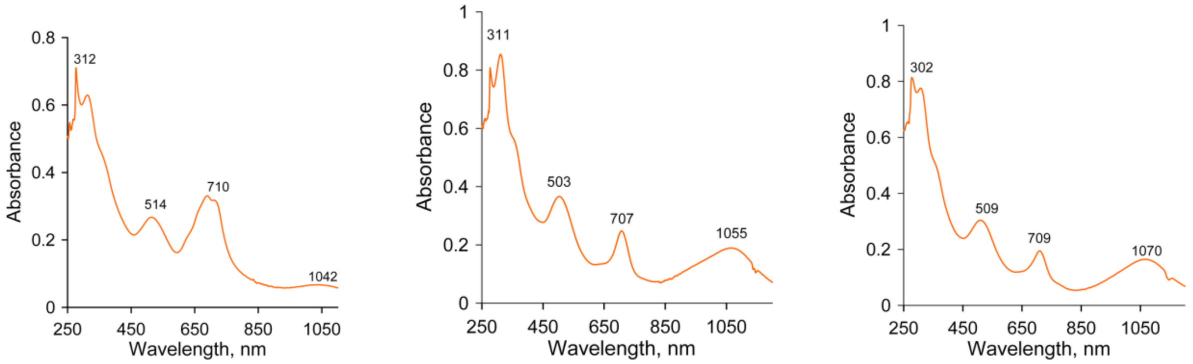

(a) (b) (c)

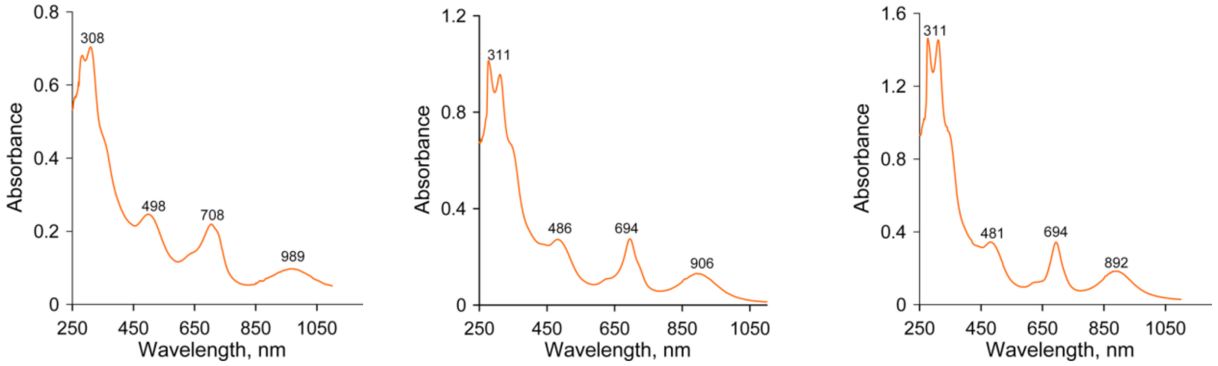

(d) (e) (f)

Figure S-2. UV-Vis spectra of the $LnPc_2$@TFA conjugates in benzene, where Ln is for (a) Nd, (b) Sm, (c) Eu, (d) Gd, (e) Yb, (f) Lu.

3. The protonation process of $LnPc_2$ complexes

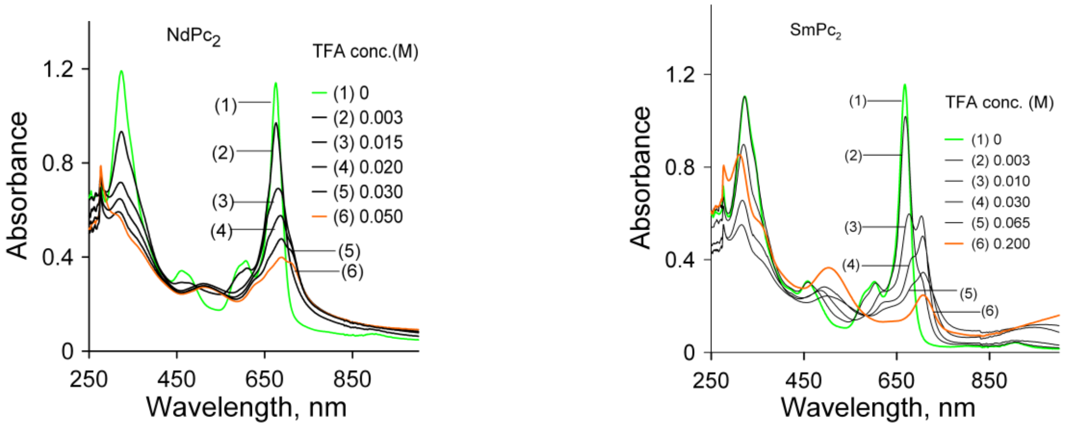

(a) (b)

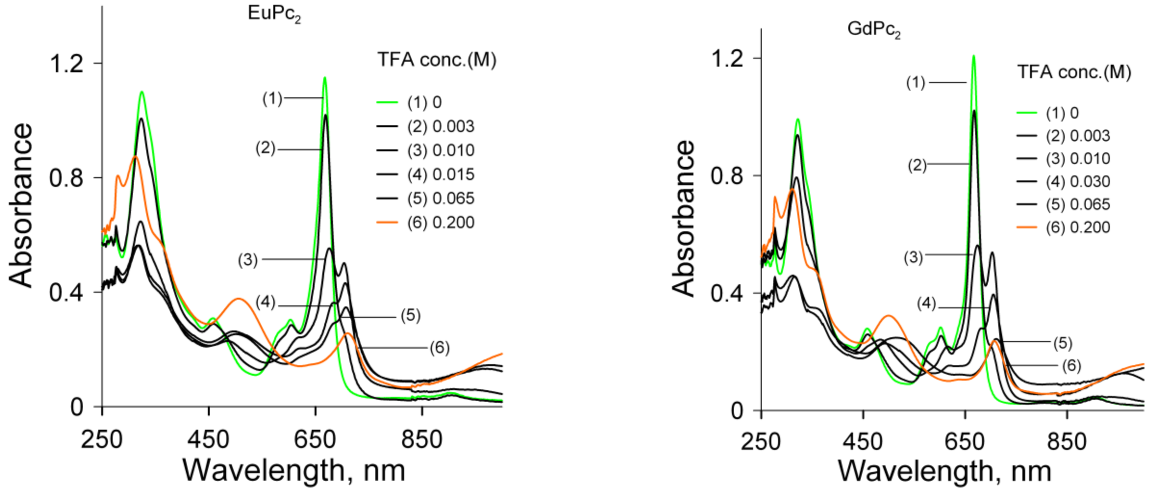

(c) (d)

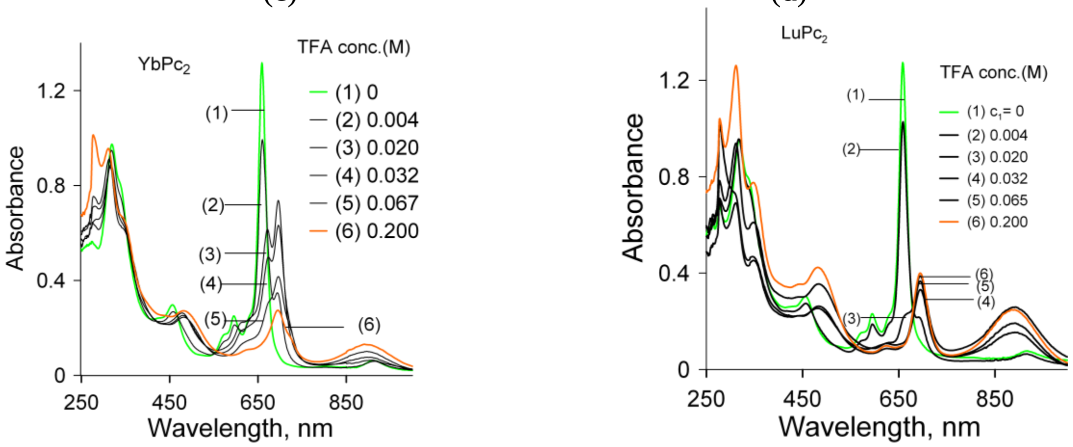

(e) (f)

Figure S-3. Progress in protonation of $LnPc_2$ by TFA in benzene monitored by UV-Vis spectra; (a) $NdPc_2$, (b) $SmPc_2$, (c) $EuPc_2$, (d) $GdPc_2$, (e) $YbPc_2$, (f) $LuPc_2$

4. Chemical stability of the $LnPc_2$@TFA conjugates

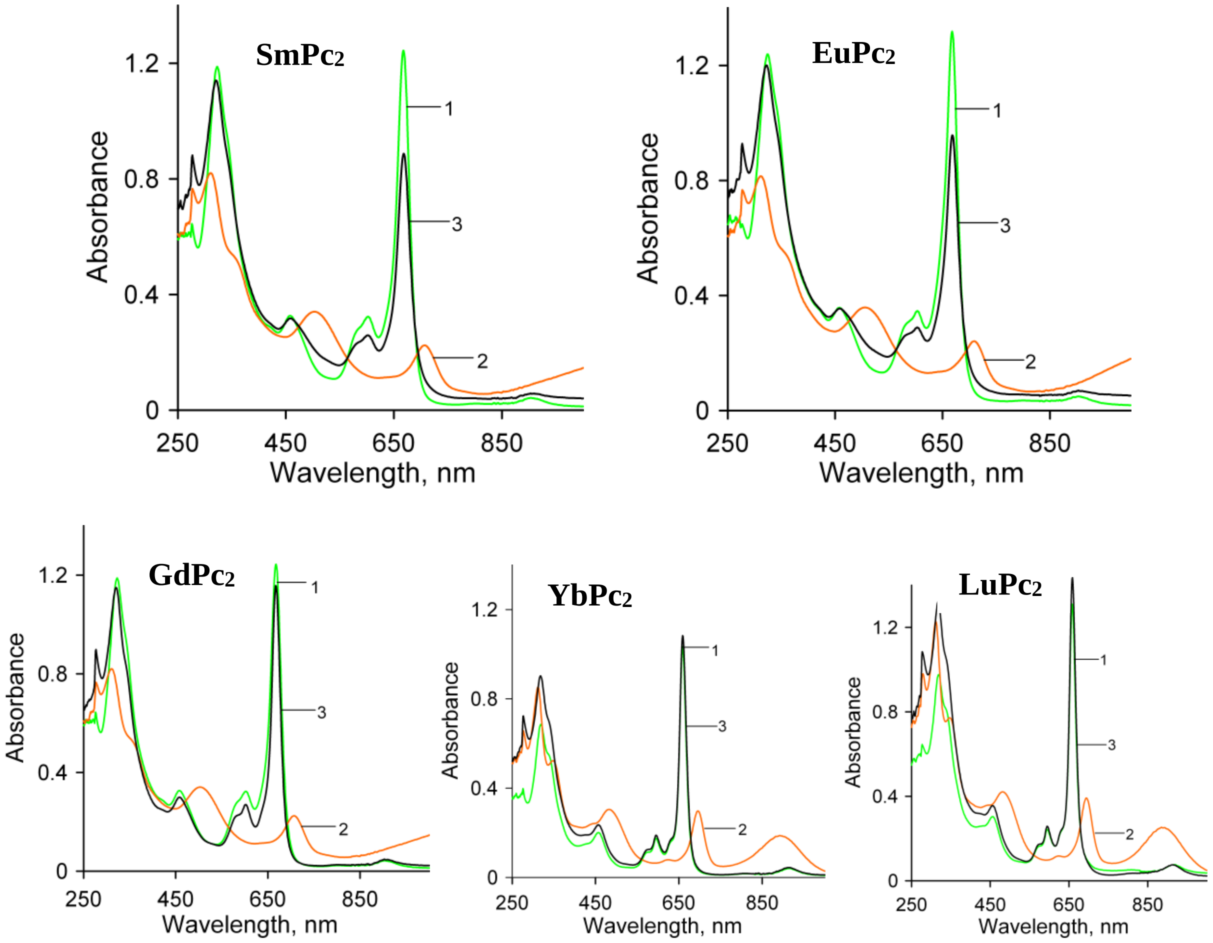

Figure S-4. The effect of TFA-protonation upon the chemical stability of $LnPc_2$ complexes manifested in the reaction of $LnPc_2$@TFA conjugates with triethylamine (TEA). The electronic absorption spectra refer to the process of recovering of the initial $LnPc_2$ compounds

from their conjugates; (1) initial form $LnPc_2$ (green line), (2) the conjugate $LnPc_2$@TFA (orange line), (3) recovery of the initial form after adding TEA to the (b) solution (black line). Note, that $NdPc_2$ completely decomposed during the reaction with TEA (hence no spectra have been shown).

5. Structural considerations based on the DFT-created model

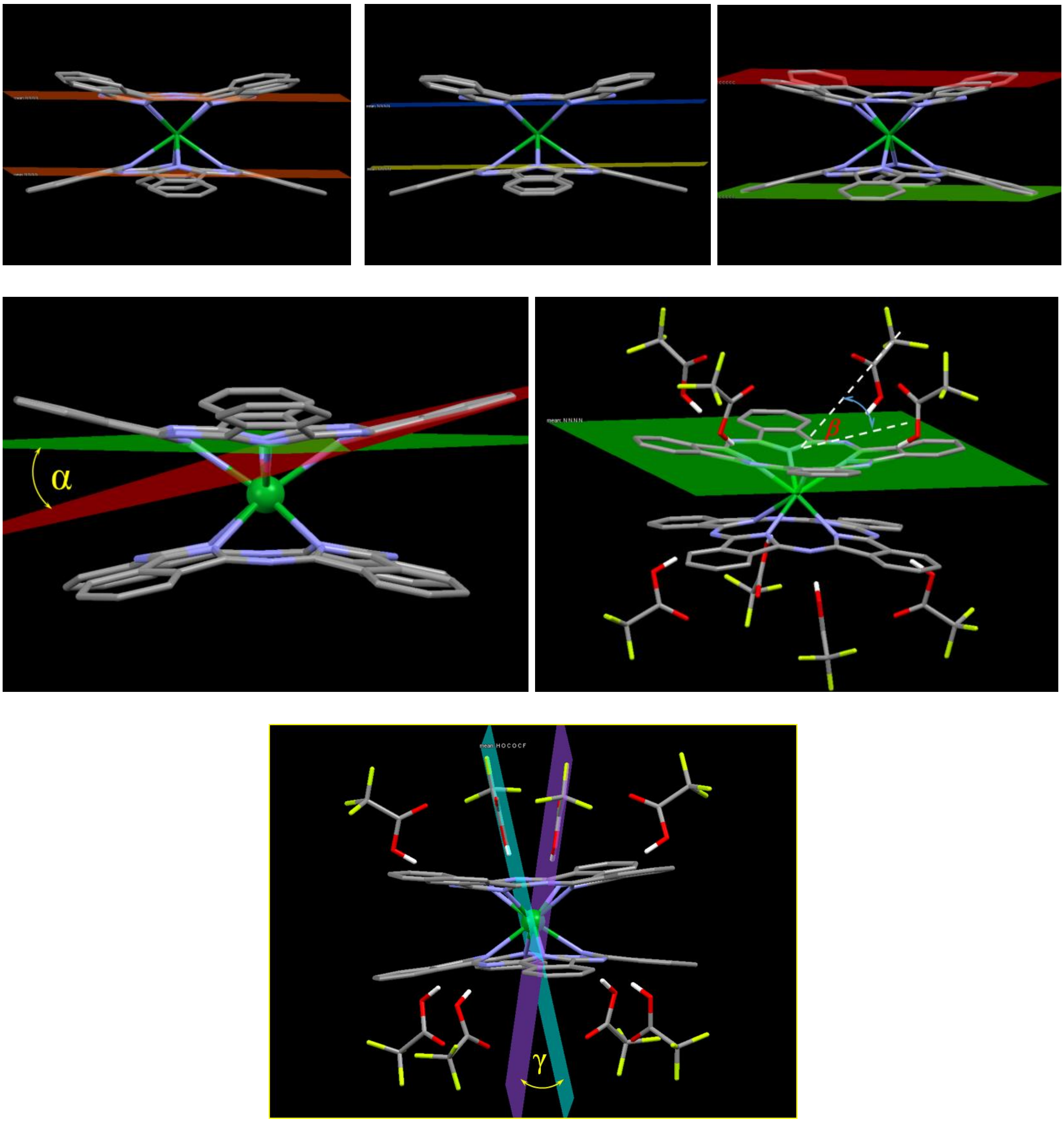

Figure S-5. Calculated molecular structures based on the $LuPc_2$ model; (a) upper row from the left, featuring the macrocycles' virtual planes : $4N_p$, $4N_\mu$ and $8C_b$ (ref. Table 3, main text); (b) below, featuring the α, β and γ angles (ref. Table 3, main text).

6. Fluorescence spectra of $LnPc_2$ and $LnPc_2$@TFA in benzene

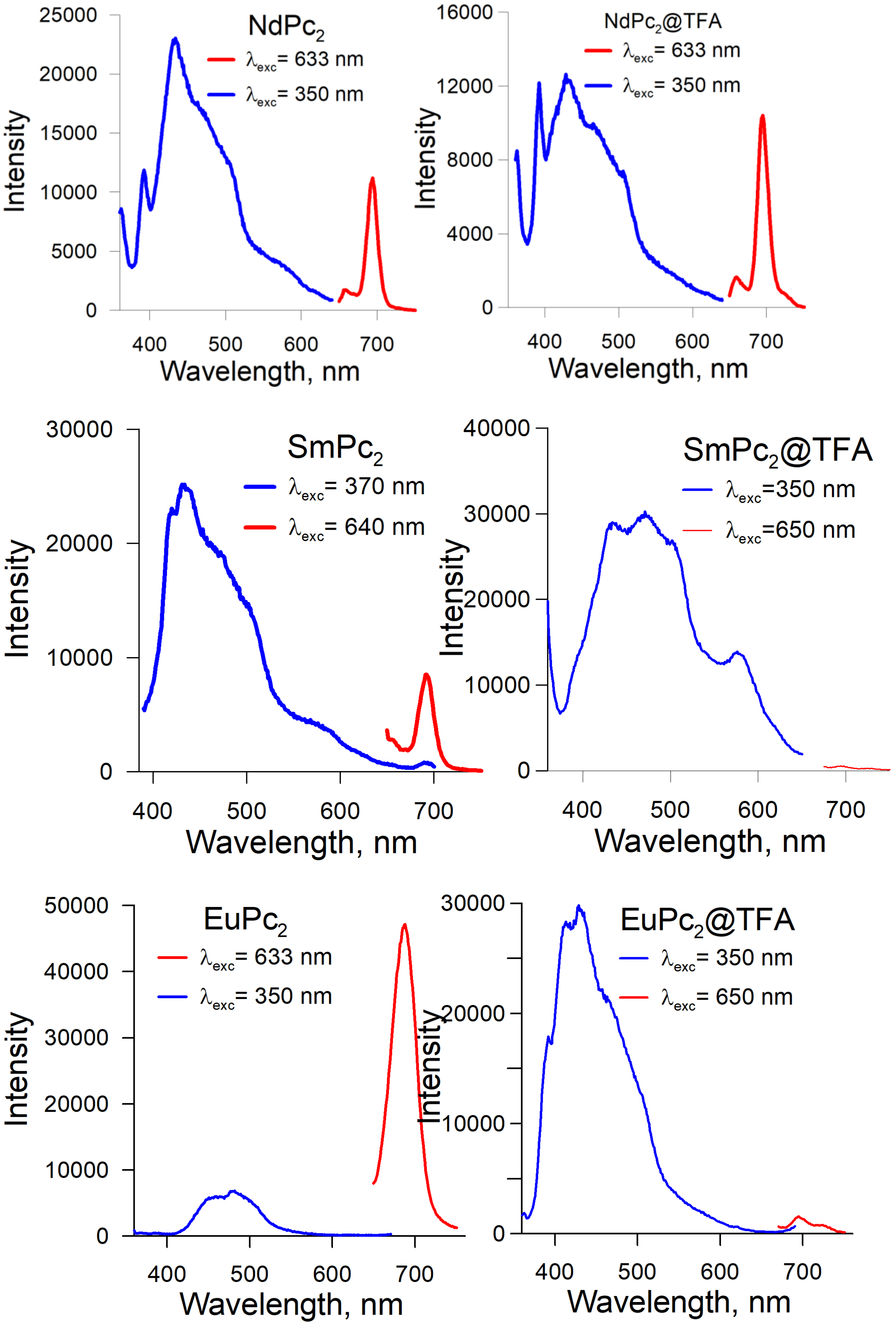

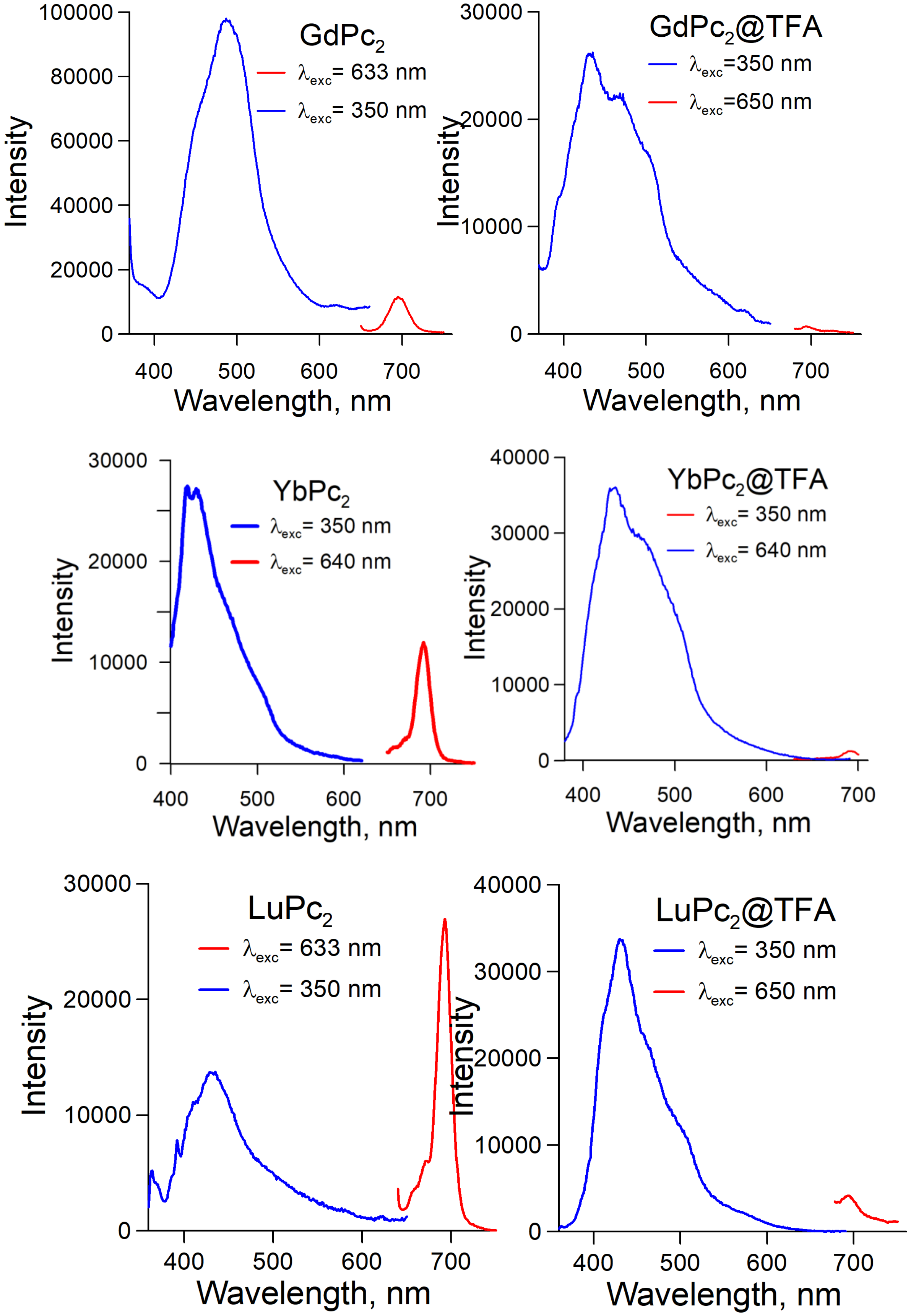

Figure S-6. Raw fluorescence emission spectra of the base $LnPc_2$ compounds and the $LnPc_2$@TFA conjugates, measured in benzene.

7. Singlet molecular oxygen related NIR emission spectra

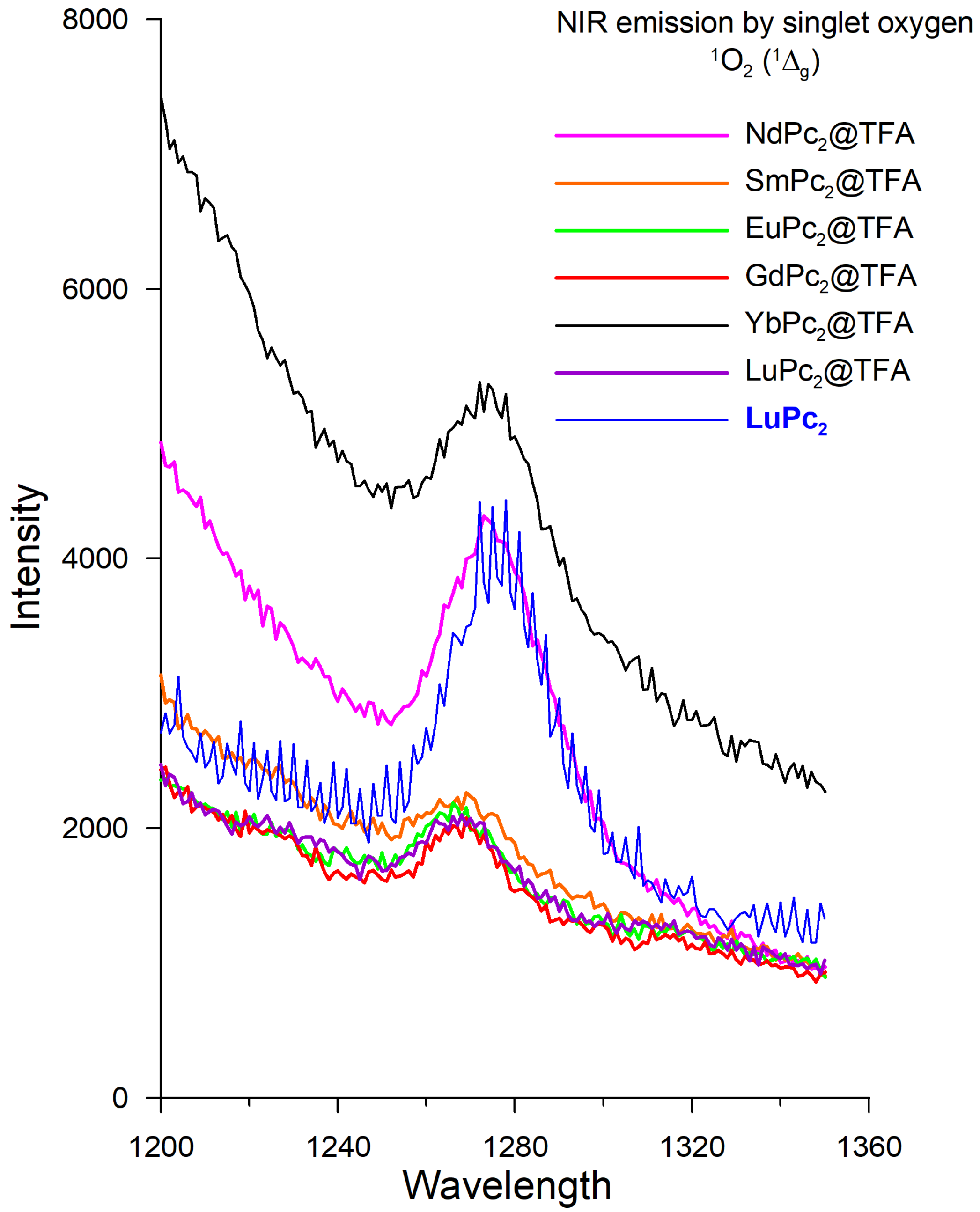

Figure S-7. Raw NIR phosphorescence emission spectra of $^1O_2$ ($^1\Delta_g$) of the $LnPc_2$@TFA conjugates and $LuPc_2$, directly measured in benzene.

8. The photodegradation process (UV-Vis spectra)

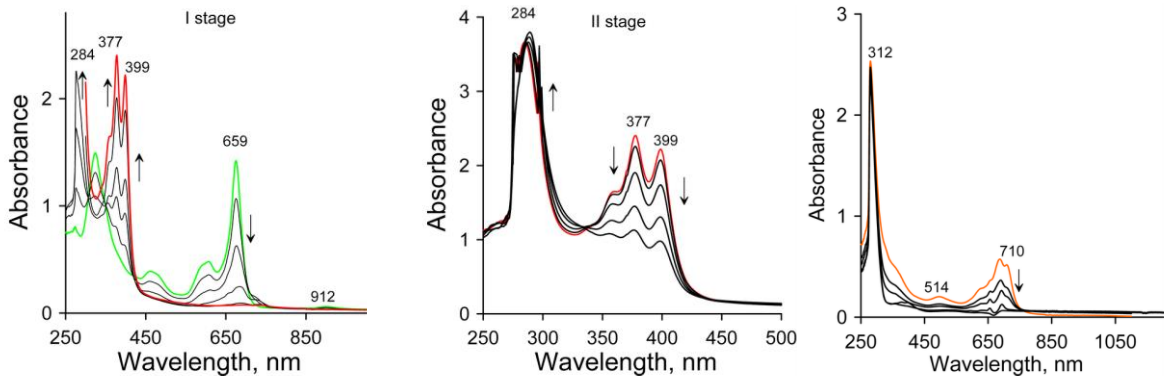

Figure S-8a. Photodegradation of $NdPc_2$ and its TFA conjugate; I stage t = 0-280 min; II stage t = 280-780 min; (right) photolysis of $NdPc_2$@TFA, t = 30 min.

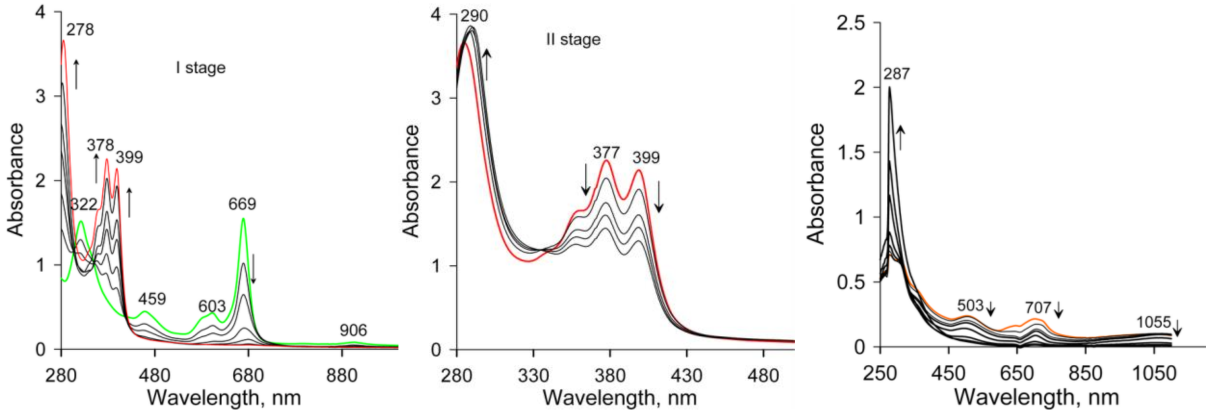

Figure S-8b. Photodegradation of $SmPc_2$ and its TFA conjugate; I stage t = 0-400 min; II stage t = 400-1200 min; (right) photolysis of $SmPc_2$@TFA, t = 100 min.

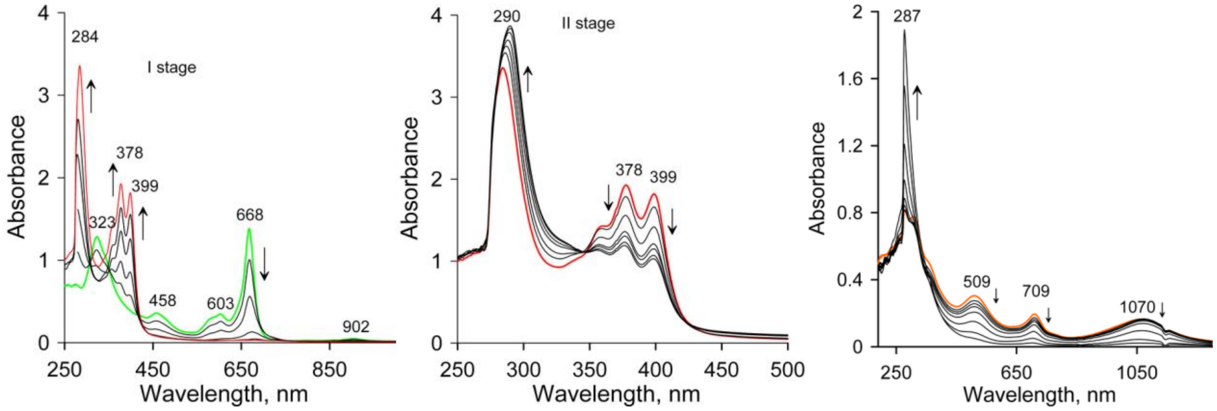

Figure S-8c. Photodegradation of $EuPc_2$ and its TFA conjugate; I stage t = 0-280 min; II stage t = 280-800 min; (right) photolysis of $EuPc_2$@TFA, t = 100 min.

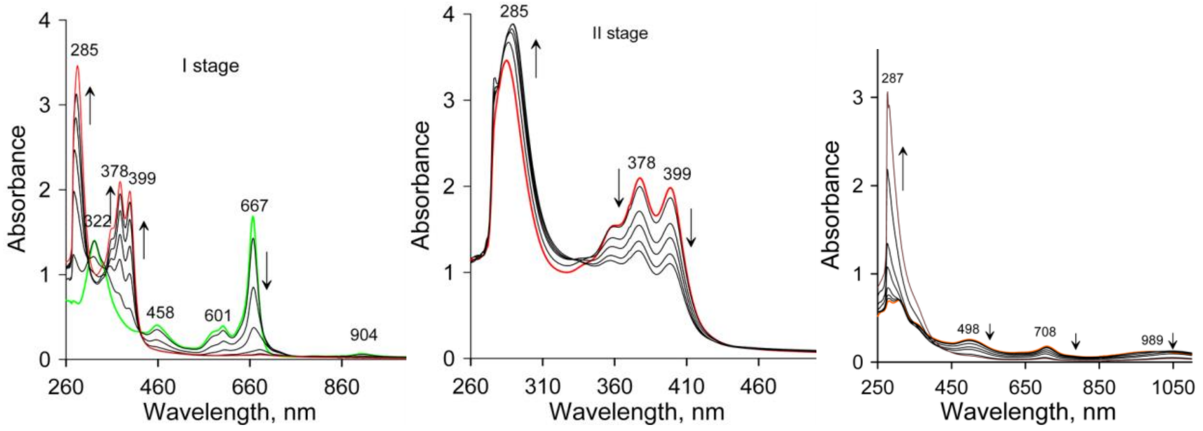

Figure S-8d. Photodegradation of $GdPc_2$ and its TFA conjugate; I stage t = 0-300 min; II stage t = 300-700 min; (right) photolysis of $EuPc_2$@TFA, t = 100 min.

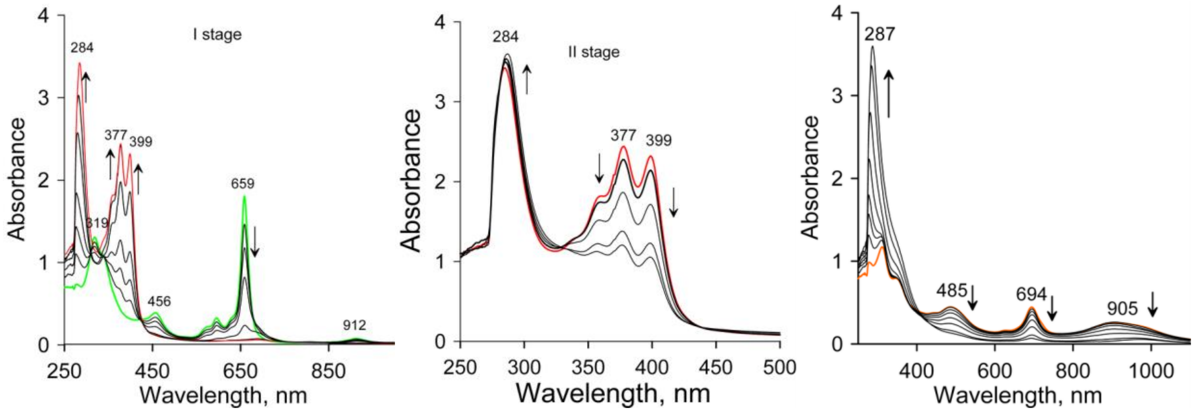

Figure S-8e. Photodegradation of $YbPc_2$ and its TFA conjugate; I stage t = 0-400 min; II stage t = 400-800 min; (right) photolysis of $YbPc_2$@TFA, t = 300 min.

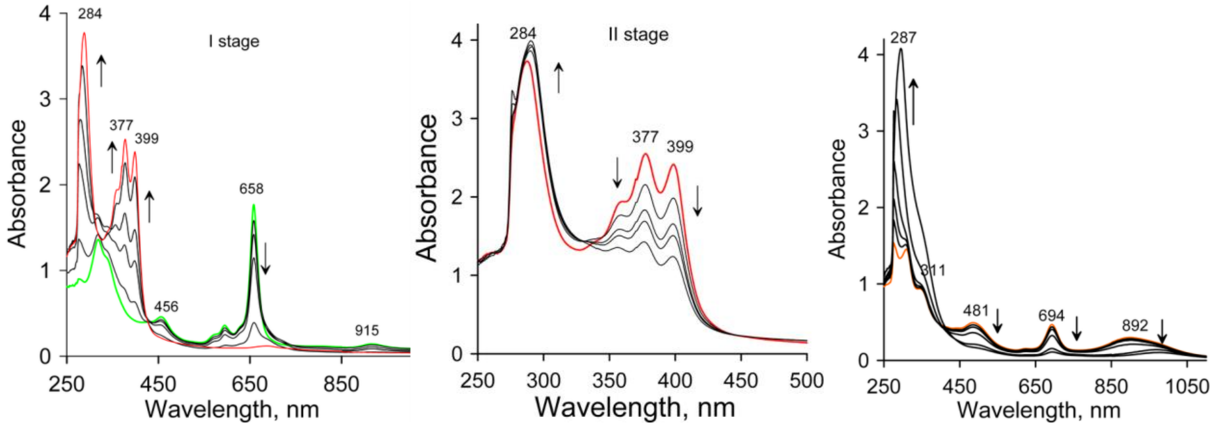

Figure S-8f. Photodegradation of $LuPc_2$ and its TFA conjugate; I stage t = 0-400 min; II stage t = 400-850 min; (right) photolysis of $LuPc_2$@TFA, t = 300 min.

9. The photodegradation process (kinetic curves)

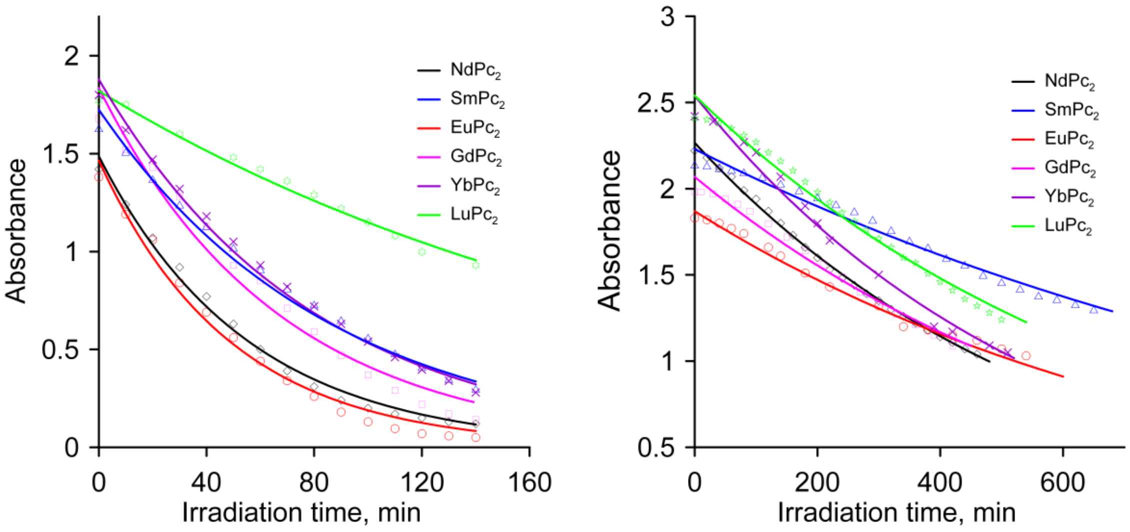

Figure S-9a. Photodegradation kinetic curves of $LnPc_2$ in benzene (ref. Table 5, main text); I stage, t = 0-140 min of UV-irradiation (left) and II stage, (degradation of the intermediate product), t =140-600 min of UV-irradiation (right).

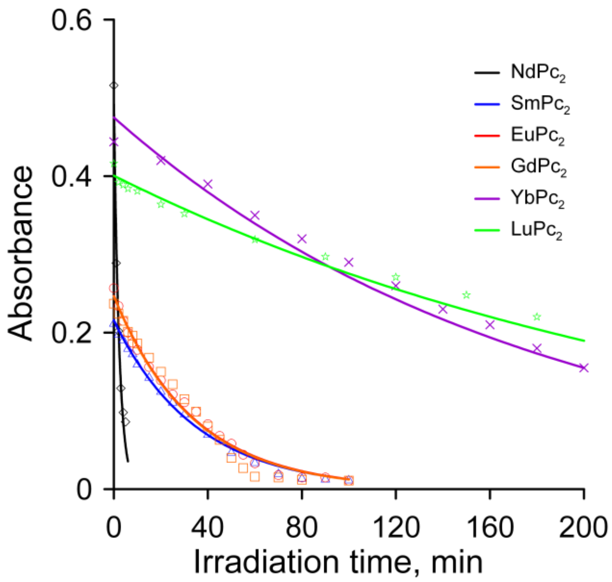

Figure S-9b. Photodegradation kinetic curves of $LnPc_2$@TFA conjugates in benzene (ref. Table 5, main text). Note the extremely fast decay of the Nd derivative.

10. Quantum yield

Table S-1. Photodegradation quantum yield (Φ) estimates. For $LnPc_2$ only the I stage was considered. Φ = # molecules decomposed / # photons absorbed; *t* – irradiation time (s)

Ln	Nd	Sm	Eu	Gd	Yb	Lu
$LnPc_2$						
t, s	16800	24000	16800	18000	24000	24000
$\Phi \times 10^3$	5.1	3.6	5.1	4.8	3.6	3.6
$LnPc_2$@TFA						
t, s	1800	6000	6000	6000	18000	18000
$\Phi \times 10^3$	45	14	14	14	4.8	4.8

$$\Phi = \frac{\Delta S \cdot N_A \cdot c \cdot h}{I_{UV} \cdot A \cdot t \cdot \lambda}$$

ΔS – number of substrate moles decomposed (approximately $4 \cdot 10^{-8}$ mol in each case)
I_{UV} – irradiance ($5 \cdot 10^{-4}$ W/cm^2)
A – surface area irradiated (2 cm^2)
t – irradiation time (s)
N_A – Avogadro constant $6.022 \cdot 10^{23}$ (mol^{-1})
c – speed of light in vacuum $3 \cdot 10^8$ (m/s)
h – Planck's constant $6.62 \cdot 10^{-34}$ (J · mol)
λ – photon vavelength (313 nm and 366 nm photons were effectively absorbed)

Comment : the quantum yields reported in Table S-1 should be considered at rough estimate only, however the calculated values seem quite reliable and are in agreement with the kinetic parameters (effective photolysis rate constants, *k*) showed in Table 5 of the main text. In other words, they do confirm the investigated compounds represent relative stable molecular systems (in particular those including the Yb^{3+} and Lu^{3+} ions).

11. The photodegradation product

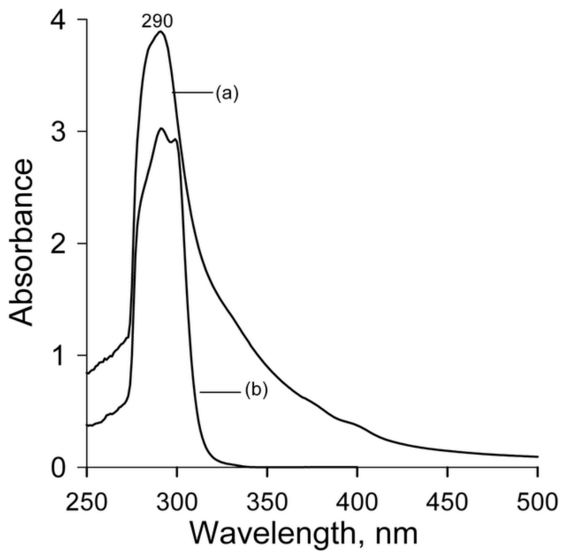

Figure S-10a. UV-Vis spectrum of the residue after photodegradation of $SmPc_2$ (a); reference spectrum of phthalimide (b); spectra were measured in benzene.

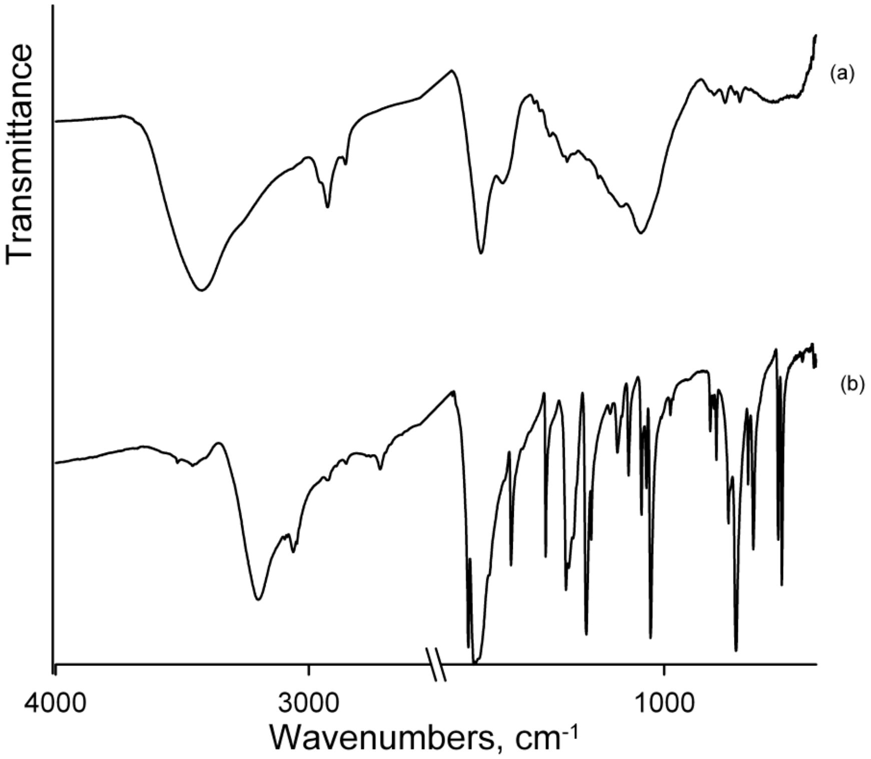

Figure S-10b. FTIR spectrum of the solid residue after photodegradation of $SmPc_2$ (a); reference spectrum of phthalimide (b); spectra were measured in a KBr pellet.
